# Primary breast diffuse large B-cell lymphoma (germinal center B-cell-like subtype): a case report and literature review

**DOI:** 10.3389/fonc.2025.1653826

**Published:** 2025-10-28

**Authors:** Xiaoxiao Xing, Daixiang Liao, Shiyun Zhang, Jun Li, Junyi Li, Ling Zhang, Yun Wang, Dongpo Zhang, Yue Wang, Yufei Li

**Affiliations:** ^1^ Department of Surgery, Guang’anmen Hospital of China Academy of Chinese Medical Sciences, Beijing, China; ^2^ Department of Pathology, Guang’anmen Hospital of China Academy of Chinese Medical Sciences, Beijing, China; ^3^ Department of Anorectal, Guang’anmen Hospital of China Academy of Chinese Medical Sciences, Beijing, China

**Keywords:** primary breast lymphoma, diffuse large B-cell lymphoma, GCB subtype, double expressor lymphoma, case report, review

## Abstract

**Background:**

Primary breast lymphoma (PBL) is a rare type of extranodal lymphoma, of which diffuse large B-cell lymphoma (DLBCL) is the most common histological subtype. Due to its nonspecific clinical and imaging features, PBL is frequently misdiagnosed as breast carcinoma or mastitis, leading to delays in treatment.

**Case Presentation:**

We report the case of a postmenopausal female who presented with a rapidly enlarging mass in the left breast and was initially misdiagnosed as non-lactational mastitis. Core needle biopsy and immunohistochemistry revealed features consistent with DLBCL of the germinal center B-cell-like (GCB) subtype. Notably, the tumor cells co-expressed Bcl-2 (95%) and c-Myc (70%), meeting the criteria for double expressor lymphoma (DEL), suggesting aggressive biological behavior. Serum lactate dehydrogenase (LDH) was markedly elevated (3902 U/L), indicating high tumor burden. Despite palliative surgery, the patient’s condition rapidly deteriorated.

**Discussion:**

GCB-type DLBCL generally carries a better prognosis than the activated B-cell-like (ABC) subtype; however, significant heterogeneity exists within the GCB subtype. DEL is recognized as a high-risk variant associated with poor outcomes, regardless of cell of origin. This case emphasizes the diagnostic challenges of PBL and highlights the need for precise immunophenotyping and individualized therapy. LDH elevation may reflect hypoxia-induced metabolic reprogramming and potential treatment resistance via the HIF-1α pathway.

**Conclusion:**

Accurate diagnosis of primary breast DLBCL requires careful differential evaluation. Immunohistochemical profiling and LDH monitoring are crucial for prognostic assessment and treatment planning. More clinical data are needed to optimize management strategies for this rare entity.

## Introduction

Diffuse large B-cell lymphoma (DLBCL) is the most common and aggressive subtype of non-Hodgkin lymphoma, characterized by significant biological heterogeneity (1). DLBCL can be further categorized into germinal center B-cell-like (GCB) and activated B-cell-like (ABC) subtypes based on gene expression profiling, with the GCB subtype generally associated with a better prognosis (2). Primary breast DLBCL (PB-DLBCL) is exceedingly rare, accounting for only 0.04% to 0.5% of all breast malignancies. It typically presents as a unilateral breast mass and is frequently misdiagnosed as breast carcinoma or non-lactational mastitis due to its nonspecific clinical features (3). The unique anatomical location and histopathological complexity of PB-DLBCL present diagnostic and therapeutic challenges. In this report, we present a case of GCB-type PB-DLBCL and provide a literature review, focusing on its differential diagnosis and the clinical implications of intragroup heterogeneity within the GCB subtype.

## Case presentation

**Figure 1:**
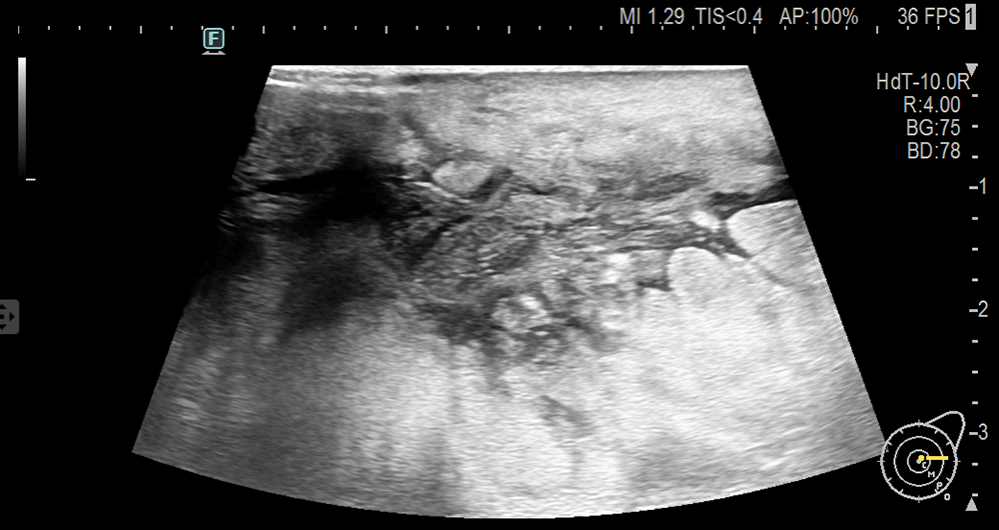
Ultrasound of breast tumor.

A 51-year-old postmenopausal woman presented with a two-month history of a progressively enlarging, painful mass in her left breast. Initially diagnosed as non-lactational mastitis at a local hospital, she received topical and systemic antibiotics without improvement. Ultrasound imaging at two different hospitals described an irregular hypoechoic mass with partial liquefaction, suggestive of inflammation. The lesion enlarged, developed ulceration, and was accompanied by skin changes. On presentation to our institution, physical examination revealed diffuse breast induration, a 3 × 3 cm ulcerated area with bloody discharge, and axillary lymphadenopathy. Laboratory results revealed elevated inflammatory markers (CRP 53.4 mg/L), mild thrombocytopenia, and significantly elevated serum LDH (3902 U/L). Ultrasound revealed a large, irregular hypoechoic area in the glandular layer of the left breast, partially extending to the skin, with unclear borders and partial anechoic liquefaction (**Figure 1**).

**Figure 2:**
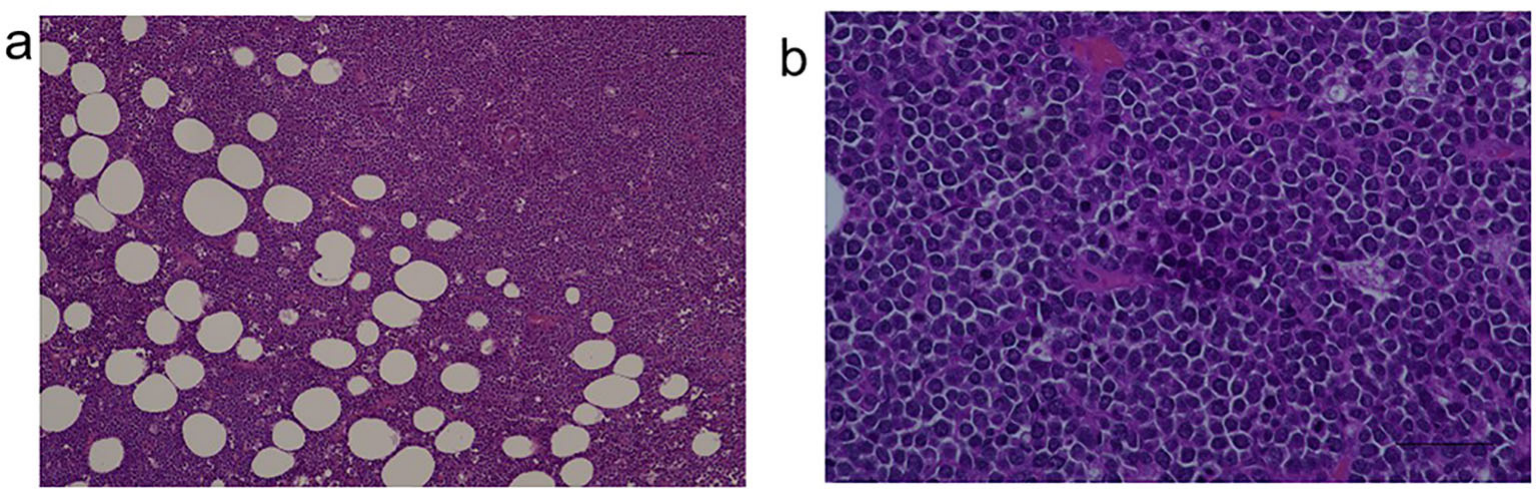
**(a)** Tumor cells and **(b)** lymphoid cells.

Ultrasound-guided core needle biopsy of the mass demonstrated diffuse infiltration of large atypical lymphoid cells(**Figures 2a, b**).

**Figure 3:**
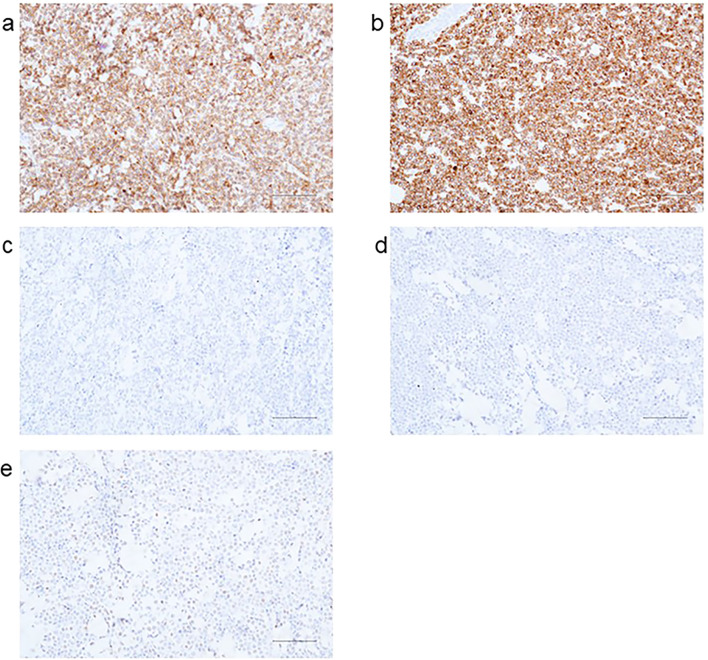
Immunohistochemical results: **(a)** CD10; **(b)** Bcl-2; **(c)** Bcl-6; **(d)** Mum-1; **(e)** C-myc.

Immunohistochemical results: AE1/AE3(-), Ki-67 (+90%), CD20(+), CD21 (FDC-), CD3 (scattered+), CD30(-), CD79α(+), CD10(+) (**Figure 3a**), Bcl-2 (+95%) (**Figure 3b**), Bcl-6 (+30%) (**Figure 3c**), Mum-1(-) (**Figure 3d**), C-myc (+70%) (**Figure 3e**), ALK (D5F3)(-); *in situ* hybridization for EBV-encoded RNA (EBER) was negative.

**Figure 4:**
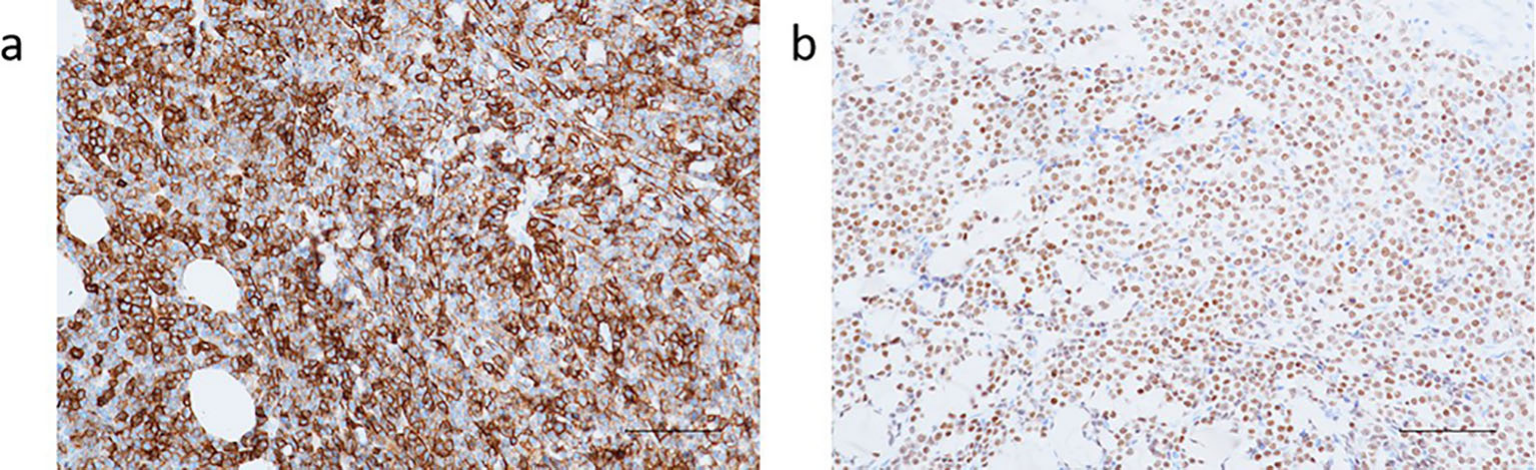
**(a)** CD20; **(b)** PAX5.

Based on the histopathology and immunophenotype, the diagnosis of germinal center B-cell-like subtype DLBCL was established (**Figures 4a, b**).

Upon admission, the left breast mass had grown to approximately 15 cm in diameter, with hyperpigmentation and increased skin temperature. A 3 × 3 cm ulcerated necrotic lesion with crusting, pain, and bloody discharge was noted near the upper areola. Multiple tender lymph nodes were palpated in the left axilla. No supraclavicular masses were found. Past medical history was unremarkable. Additional lab results: ALT 55.8 U/L (7–40), AST 57.7 U/L (13–35), LDH 3902 U/L (<247), albumin 34.6 g/L (40–55), WBC 9.43×10^9^/L, neutrophils 5.8×10^9^/L, CRP 53.4 mg/L, platelets 109×10^9^/L. During hospitalization, swelling and redness of the left breast worsened. On day 3, the patient developed tachycardia (up to 130 bpm), cold sweating, and hypotension. Labs: CRP 70.10 mg/L, D-dimer 2.84 mg/L FEU (0–0.55), FDP 5.2 mg/L (0–5), fibrinogen 4.40 g/L (2–4), procalcitonin 0.23 ng/mL (0–0.1). A small pericardial effusion was also observed. Supportive care included ECG monitoring, oxygen, antibiotics, anticoagulation, albumin, and diuretics. ultidisciplinary consultation concluded that chemotherapy was first-line treatment, but the patient’s rapidly progressing disease and general deterioration made it temporarily unfeasible. Palliative surgery was recommended to reduce tumor burden and inflammation, potentially creating a window for subsequent therapy. With informed consent, the patient underwent left total mastectomy on November 21, 2024. Postoperative management included ECG monitoring, anti-infection therapy, albumin supplementation, and platelet elevation. Post-op labs: WBC 7.17×10^9^/L, hemoglobin 76 g/L, platelets 80×10^9^/L, CRP 88.23 mg/L. On postoperative day 3, the patient experienced sudden cardiopulmonary arrest. Cardiopulmonary resuscitation, intubation, and defibrillation were performed. Spontaneous respiration and pulse were restored, and she was transferred to the ICU. One month later, the patient died of multi-organ failure and hypoxic-ischemic encephalopathy due to rapid disease progression. Her family expressed gratitude for the timely diagnosis, treatment, and rescue efforts.

## Discussion

Primary breast lymphoma (PBL) is a rare form of extranodal lymphoma characterized by initial localization within the breast tissue, with or without involvement of regional lymph nodes and without evidence of extramammary disease. Histologically, PBL encompasses various subtypes, including diffuse large B-cell lymphoma (DLBCL), mucosa-associated lymphoid tissue (MALT) lymphoma, follicular lymphoma, Burkitt lymphoma, and anaplastic large cell lymphoma. Among these, DLBCL is the most prevalent subtype (4). Clinically, PB-DLBCL often presents as a unilateral breast mass, with the right breast more commonly affected than the left. Ipsilateral axillary lymph node enlargement is observed in approximately 30–40% of cases (5, 6). Due to the lack of specific radiological features, definitive diagnosis relies heavily on pathological examination and immunohistochemistry. PB-DLBCL typically demonstrates diffuse infiltration by large atypical B cells and an immunophenotypic profile that includes positivity for CD20 and PAX5, and negativity for CD3 (4, 7). Currently, there is no universally accepted standard treatment protocol for PB-DLBCL. The most widely adopted first-line approach involves chemoimmunotherapy based on the R-CHOP regimen, which includes rituximab, cyclophosphamide, doxorubicin, vincristine, and prednisone (8). A study has shown that patients with DLBCL achieve excellent outcomes after receiving rituximab-containing immuno-chemotherapy; however, patients with DEL respond poorly to the standard R-CHOP regimen, with non-GCB subtype exhibiting worse clinical outcomes, and some patients developing resistance to the CD20 monoclonal antibody (rituximab) (9). The DA-EPOCH-R regimen, as a dose-adjusted, intensified chemoimmunotherapy regimen, utilizes continuous intravenous infusion of chemotherapeutic agents to increase tumor cell exposure time to the drugs, thereby enhancing the anti-tumor efficacy. Research indicates that for DEL patients, the DA-EPOCH-R regimen can achieve a complete response rate of 65%-70% and a 3-year survival rate of approximately 55%, which is significantly superior to the R-CHOP regimen (10). Its advantage lies in effectively inhibiting MYC and Bcl-2 mediated cell proliferation and anti-apoptotic signals, making it particularly suitable for DEL patients with high tumor burden (elevated LDH), as in the present case (11). However, the toxic side effects of this regimen, such as myelosuppression, are more pronounced, requiring close monitoring of complete blood count and hepatic/renal function, and it should be used cautiously in patients with poor overall condition (12, 13). Some patients may also receive radiotherapy or surgery depending on disease extent and response to chemotherapy. Compared with breast carcinoma, PB-DLBCL tends to be more responsive to chemotherapy. Therefore, the role of surgery is relatively limited and often restricted to diagnostic biopsy or breast-conserving procedures, while radical mastectomy—commonly used in breast cancer—is generally not required (14).

DLBCL is essentially a group of malignant lymphomas with a high degree of molecular and clinical heterogeneity. Based on Gene Expression Profiling (GEP) analysis, Alizadeh et al. classified DLBCL for the first time into two major molecular subtypes: Germinal Center B-cell-like (GCB) and Activated B- cell-like (ABC) type (1). Among them, GCB-type tumors usually express CD10 and Bcl-6 and are negative for MUM1/IRF4 expression, while ABC-type exhibits post-germinal center features such as MUM1 positivity and CD10 negativity (2). Although the overall prognosis of GCB type is better than that of ABC type, recent studies have shown that even within the GCB type of DLBCL, significant biological heterogeneity still exists, which has a profound impact on treatment response and survival outcomes (15). The case presented in this study exemplifies this combination of phenotypically uniform and functionally diverse features: the patient was molecularly characterized as a GCB-type DLBCL, but also possessed the features of Double Expressor Lymphoma (DEL), and had a significantly elevated serum LDH level, suggesting a more aggressive biological behavior of the tumor. DEL is defined as the expression of both Bcl-2 and c-Myc proteins at high levels in tumor cells. Although this expression pattern can occur in GCB or ABC types, it is strongly associated with a poor prognosis regardless of its origin (15, 16). DEL is different from “double-hit lymphoma” (DHL), which refers to lymphoma with BCL2, MYC or BCL6 gene rearrangements, but DEL is also predictive of tumors with high metabolic activity, inhibition of apoptosis, and risk of treatment resistance.

In this case, the patient presented with a rapidly progressing breast mass, which was initially misdiagnosed multiple times as mastitis. The International Prognostic Index (IPI) score was 2 (17). Further evaluation revealed immunohistochemical features of CD10 positivity, partial Bcl-6 positivity (30%), and MUM1 negativity, consistent with a GCB subtype (18). Additionally, the patient was EBER-negative, ruling out EBV-related disease. Notably, the tumor also exhibited a double expressor lymphoma (DEL) phenotype, with Bcl-2 expression at 95% and c-Myc expression at 70%, suggesting aggressive biological behavior that may have contributed to the rapid disease progression (19). Due to the rarity of PB-DLBCL and the nonspecific imaging features, diagnosis is often challenging, particularly in distinguishing it from breast carcinoma and non-lactational mastitis. Non-lactational mastitis predominantly occurs in younger women, whereas PB-DLBCL is more common in postmenopausal women. Both conditions can present with irregularly shaped lesions, spiculated margins, and heterogeneous internal echoes on ultrasound (20), making it difficult to differentiate them radiologically. However, non-lactational mastitis is usually associated with elevated white blood cell count and CRP levels, and shows significant improvement after antibiotic treatment, whereas PB-DLBCL often lacks significant laboratory abnormalities and responds poorly to anti-inflammatory or antibiotic therapy. When history, physical examination, and laboratory findings do not yield a clear diagnosis, core needle biopsy and pathological analysis should be promptly performed (21). PB-DLBCL should also be differentiated from breast carcinoma. While breast cancer typically presents as a painless mass, often accompanied by nipple retraction, peau d’orange, or nipple discharge (22), PB-DLBCL usually presents as a rapidly enlarging painless mass over a short period and lacks those characteristic signs. On imaging, PB-DLBCL typically appears as a patchy hypoechoic area with rich blood flow signals and minimal calcification, rarely showing spiculated margins (23, 24). In contrast, breast cancer often presents as an irregular hypoechoic mass with clustered microcalcifications and spiculated borders (25). Thus, definitive diagnosis of PB-DLBCL still relies on histopathological and immunohistochemical confirmation.

Clinical misdiagnosis of PB-DLBCL as non-puerperal mastitis or breast cancer leads to a completely divergent treatment pathway. Antibiotic and anti-inflammatory therapies for non-puerperal mastitis are ineffective against lymphoma, which not only wastes medical resources but also allows for rapid tumor progression during an ineffective treatment period. In this case, a two-month course of antibiotic therapy was administered, during which the mass continued to enlarge with subsequent ulceration. The tumor burden increased significantly (LDH reached 3902 U/L), and the optimal window for intervention was missed. This diagnostic delay increased subsequent treatment risks: the progression of the disease led to an expanded tumor invasion (involving the skin and axillary lymph nodes) and triggered severe metabolic disturbances and inflammatory responses. Consequently, the patient was deprived of the opportunity for initial chemotherapy and could only undergo palliative surgery, which was associated with a significantly elevated risk of postoperative complications. Furthermore, the prognosis was markedly worsened. The therapeutic efficacy for PB-DLBCL is highly dependent on early intervention. A study has shown that a treatment delay of more than one month due to misdiagnosis is associated with an approximately 30% reduction in the 5-year survival rate (26). In this case, the patient ultimately died of multi-organ failure, which was closely related to the disease progression attributable to the initial misdiagnosis.

To further understand the clinical implications of this patient’s molecular profile, we reviewed the characteristics of DEL and its associated molecular mechanisms. DEL is defined as high co-expression of Bcl-2 and c-Myc proteins on immunohistochemistry, present in approximately 15% to 20% of GCB-type DLBCL cases (16). MYC gene rearrangements are found in approximately 5% to 15% of DLBCL cases, while BCL2 rearrangements are more common, occurring in about one-third of cases, mainly within the GCB subtype (27). Importantly, overexpression of BCL2 is not solely attributable to gene translocation. In the ABC subtype of DLBCL, BCL2 gene amplification is observed in approximately two-thirds of cases, which may represent an alternative mechanism of overexpression. Some studies have shown that if BCL2 rearrangement is not accompanied by MYC rearrangement and is the sole chromosomal abnormality, it may not significantly impact survival (28, 29). Currently, the prognostic impact of BCL2 expression in different DLBCL subtypes remains controversial, especially in the context of DEL (30).

The development of next-generation sequencing (NGS) technology has facilitated more precise genetic subclassification of DLBCL. Schmitz et al. identified four major genetic subtypes: MCD (co-occurrence of MYD88 and CD79B mutations), BN2 (BCL6 fusions and NOTCH2 mutations), N1 (NOTCH1 mutations), and EZB (EZH2 mutations and BCL2 rearrangement). The EZB subtype typically originates from the GCB lineage and is strongly associated with BCL2-related alterations. These subtypes show distinct mutational profiles, pathway activity, and variable responses to immunochemotherapy (15). In clinical practice, the application of NGS molecular subtyping includes refining prognostic stratification. Traditional immunohistochemistry-based classification often fails to fully capture tumor heterogeneity, whereas NGS allows for the identification of high-risk subgroups through the detection of key gene mutations. For example, among patients with GCB-type DLBCL, those belonging to the EZB subtype who also harbor a DEL phenotype exhibit a significantly poorer prognosis compared to those without EZB alterations, thereby providing a basis for precise risk assessment (31). If NGS had been performed preoperatively in this case, it might have yielded the following critical information: determining the presence of EZH2 mutations and BCL2 rearrangements to confirm EZB subtype classification and further validate the molecular basis of the unfavorable prognosis; detecting rearrangements in MYC and BCL2 genes to exclude the possibility of DHL and clarify the molecular drivers of the DEL phenotype; and screening for mutations in HIF-1α pathway-related genes to elucidate the cause of markedly elevated LDH levels, thus offering potential directions for subsequent targeted therapy. Therefore, in GCB-type PB-DLBCL cases exhibiting DEL phenotype along with EZB-related mutations, the prognosis may be worse, and more precise treatment approaches may be warranted.

In addition to molecular features, this patient demonstrated significant metabolic abnormalities, with a serum lactate dehydrogenase (LDH) level of 3902 U/L, far exceeding the normal range. LDH, a key enzyme in glycolysis, is widely distributed in various tissues, particularly in metabolically active tumor cells. Elevated LDH typically reflects increased tumor burden and high metabolic activity (32). In DLBCL, LDH is a key parameter in the IPI scoring system and is associated with therapeutic response, relapse risk, and overall survival (33). Furthermore, the DEL phenotype itself constitutes an independent poor prognostic factor. Studies indicate that GCB-type DLBCL patients exhibiting both the DEL phenotype and elevated LDH have a 2-year survival rate of only 35%-40%, which is substantially lower than that of patients with only a single risk factor (approximately 60%-70%) (34), the clinical outcome observed in this case is consistent with this pattern. Biologically, rapid tumor proliferation often leads to local hypoxia, which stabilizes hypoxia-inducible factor-1 alpha (HIF-1α), thereby activating downstream signaling pathways (35). HIF-1α enhances reliance on anaerobic glycolysis, further increasing LDH levels, and promotes angiogenesis (via VEGF upregulation), inhibits apoptosis (via BNIP3 regulation), and contributes to tumor stemness and invasiveness. These mechanisms collectively confer resistance to chemotherapy and immunotherapy (36, 37). In this case, despite undergoing palliative surgery, the patient experienced rapid tumor progression, which corroborates the therapeutic dilemma resulting from this combination of features. Several studies have shown that elevated LDH is associated with poor initial response, higher relapse rates, and reduced overall survival in DLBCL. Therefore, LDH is not only a simple biomarker of tumor burden but also a potential indicator of metabolic reprogramming and treatment resistance (33, 38). In the future, patients with DEL phenotype and markedly elevated LDH levels may benefit from combination therapies that include HIF-1α inhibitors or targeted interventions against tumor metabolism.

Patients with DEL are known to respond poorly to standard R-CHOP therapy. Some studies have proposed the use of intensified regimens such as DA-EPOCH-R or dose-adjusted R-EPOCH to improve clinical outcomes (19). Moreover, the role of radiotherapy and surgical intervention following chemotherapy in PB-DLBCL remains uncertain. Treatment strategies should be individualized based on tumor biology, disease extent, and patient preferences (3, 14).

## Conclusion

PB-DLBCL is a rare and biologically heterogeneous lymphoma that requires a high index of suspicion for diagnosis and an individualized approach to treatment. This report details a rare case of GCB-subtype PB-DLBCL with a DEL phenotype and markedly elevated LDH, thereby contributing clinical data on such rare presentations and documenting the complete diagnostic, therapeutic, and prognostic course. It serves as an instructive example for enhancing clinical understanding of this disease. Through an in-depth case-based analysis, the clinical impact of misdiagnosis and key points for differential diagnosis are discussed. The synergistic adverse prognostic effect of the DEL phenotype combined with high LDH is explored from a mechanistic perspective, offering practical insights for avoiding misdiagnosis and improving prognostic evaluation. Furthermore, this case highlights the complexity of coexisting GCB subtype and DEL phenotype with markedly elevated LDH, indicating that traditional classification may not fully capture the tumor’s biological behavior. Further multi-omics studies focusing on the molecular and metabolic characteristics of PB-DLBCL are warranted to improve early diagnosis, identify high-risk patients, and develop more precise and effective treatment strategies. As a single-case report, this study has inherent limitations, including a small sample size and the inability to perform statistical analyses. The conclusions drawn require validation through larger, multi-center case series.

## Data Availability

The original contributions presented in the study are included in the article/supplementary material. Further inquiries can be directed to the corresponding author.

## Glossary

PBL: Primary Breast Lymphoma
DLBCL: Diffuse Large B-Cell Lymphoma
GCB: Germinal Center B-cell-like
DEL: Double Expressor Lymphoma
LDH: Lactate Dehydrogenase
ABC: Activated B-Cell-like
PB-DLBCL: Primary Breast Diffuse Large B-Cell Lymphoma
EBER: EBV-Encoded RNA
EBV: Epstein-Barr Virus
WBC: White Blood Cell
CRP: C-Reactive Protein
ALT: Alanine Aminotransferase
AST: Aspartate Aminotransferase
ECG: Electrocardiogram
FDP: Fibrin Degradation Products
FEU: Fibrinogen Equivalent Units
IPI: International Prognostic Index
HIF-1α: Hypoxia-Inducible Factor 1-alpha
VEGF: Vascular Endothelial Growth Factor
BNIP3: BCL2/adenovirus E1B 19 kDa protein-interacting protein 3
NGS: Next Generation Sequencing
MCD: MYD88 and CD79B Co-mutated Subtype
BN2: BCL6 Fusion and NOTCH2 Mutated Subtype
N1: NOTCH1 Mutated Subtype
EZB: EZH2 Mutated and BCL2 Rearranged Subtype
DHL: Double-Hit Lymphoma
R-CHOP: Rituximab, Cyclophosphamide, Doxorubicin, Vincristine, and Prednisone
DA-EPOCH-R: Dose-Adjusted Etoposide, Prednisone, Vincristine, Cyclophosphamide, Doxorubicin, and Rituximab.
